# Re: Modified Oxymatrine as Novel Therapeutic Inhibitors Against Monkeypox and Marburg Virus Through Computational Drug Design Approaches

**DOI:** 10.1111/jcmm.70266

**Published:** 2024-12-04

**Authors:** Hinpetch Daungsupawong, Viroj Wiwanitkit

**Affiliations:** ^1^ Private Academic Consultant Phonhong Lao People's Democratic Republic; ^2^ Department of Research Analytics, Saveetha Dental College and Hospitals Saveetha Institute of Medical and Technical Sciences Chennai India

Dear Editor,

We would like to comment on the publication ‘Modified oxymatrine as novel therapeutic inhibitors against Monkeypox and Marburg virus through computational drug design approaches [1]’. This work raises an essential and topical concern about the global impact of viral illnesses, specifically monkeypox and Marburg viruses, emphasising the critical need for effective therapeutic interventions. The choice of oxymethrin as the main ingredient because of its alkaloid characteristics is a good first step. While the alterations made to the oxymethrin derivatives appear novel, full explanations of the specific functional group selections used are missing. What criteria are utilised to assess the suitability and relevance of these functional groups for improving treatment activity against the mpox and Marburg viruses? Additional context should be provided regarding the relationship between functional group changes and reported increases in binding affinity. A clear explanation of the rationale for these choices will substantially improve the scientific rigour.

Computational approaches such as PASS prediction, molecular docking and ADMET profiling are useful and widely employed in drug discovery. However, there are worries regarding the predictive models' dependability. Could the authors, for example, go into more detail about the computational model validation process? What standard or experimentally validated data sets were used to support the predictions? Furthermore, knowledge about how the molecular dynamics simulations were set up may increase reproducibility. A more robust strategy would involve investigating consensus scoring approaches that combine several computational methodologies. Together, they give greater support for forecasting effective medication candidates.

In terms of future directions, exploring synergistic combinations of oximethrin derivatives with known antiviral agents may be novel, given the increasing threat of viral outbreaks. Investigation of combination therapies may enhance treatment efficacy and reduce potential resistance. Furthermore, elucidating the molecular mechanisms of action through in vitro and in vivo studies will be essential to understand the biological effects of the resulting modifications. Future studies may focus on comprehensively assessing the pharmacological effects of these derivatives, including their toxicity, therapeutic index and clinical translation potential.

Finally, although computational analysis of studies is an important step in drug development, translating the findings to real‐world efficacy requires careful animal modelling or clinical trials. The authors should emphasise the importance of integrating experimental validation to validate the obtained computational results. Bridging the gap between in silico findings and concrete clinical applications is essential for the development of novel drugs against mycoplasma and Marburg virus to ensure that they effectively address the pressing public health challenges posed by these viral diseases.

## Author Contributions

**Hinpetch Daungsupawong:** conceptualization (equal), validation (equal), visualization (equal), writing – original draft (equal), writing – review and editing (equal). **Viroj Wiwanitkit:** conceptualization (equal), supervision (equal), validation (equal), visualization (equal).

## Conflicts of Interest

The authors declare no conflicts of interest.

## Data Availability

There is no new data generated.
